# miRNA-337-3p suppresses neuroblastoma progression by repressing the transcription of matrix metalloproteinase 14

**DOI:** 10.18632/oncotarget.4311

**Published:** 2015-06-15

**Authors:** Xuan Xiang, Hong Mei, Xiang Zhao, Jiarui Pu, Dan Li, Hongxia Qu, Wanju Jiao, Jihe Zhao, Kai Huang, Liduan Zheng, Qiangsong Tong

**Affiliations:** ^1^ Department of Pediatric Surgery, Union Hospital, Tongji Medical College, Huazhong University of Science and Technology, Wuhan, Hubei Province, P. R. China; ^2^ Department of Pathology, Union Hospital, Tongji Medical College, Huazhong University of Science and Technology, Wuhan, Hubei Province, P. R. China; ^3^ Burnett School of Biomedical Sciences, College of Medicine, University of Central Florida, Orlando, Florida, USA; ^4^ Clinical Center of Human Genomic Research, Union Hospital, Tongji Medical College, Huazhong University of Science and Technology, Wuhan, Hubei Province, P. R. China

**Keywords:** neuroblastoma, microRNA-337-3p, matrix metalloproteinase 14, transcriptional repression

## Abstract

Recent evidence shows the emerging roles of endogenous microRNAs (miRNAs) in repressing gene transcription. However, the miRNAs inhibiting the transcription of matrix metalloproteinase 14 (*MMP-14*), a membrane-anchored MMP crucial for the tumorigenesis and aggressiveness, still remain largely unknown. In this study, through mining computational algorithm program and genome-wide Argonaute profiling dataset, we identified one binding site of miRNA-337-3p (miR-337-3p) within the *MMP-14* promoter. We demonstrated that miR-337-3p was under-expressed and inversely correlated with MMP-14 expression in clinical specimens and cell lines of neuroblastoma (NB), the most common extracranial solid tumor in childhood. Patients with high miR-337-3p expression had greater survival probability. miR-337-3p suppressed the promoter activity, nascent transcription, and expression of *MMP-14*, resulting in decreased levels of vascular endothelial growth factor, in cultured NB cell lines. Mechanistically, miR-337-3p recognized its binding site and recruited Argonaute 2 to facilitate the enrichment of repressive epigenetic markers and decrease the binding of RNA polymerase II and specificity protein 1 on the *MMP-14* promoter. Gain- and loss-of-function studies demonstrated that miR-337-3p suppressed the growth, invasion, metastasis, and angiogenesis of NB cells *in vitro* and *in vivo*. In addition, restoration of MMP-14 expression rescued the NB cells from changes in these biological features. Taken together, these data indicate that miR-337-3p directly binds the *MMP-14* promoter to repress its transcription, thus suppressing the progression of NB.

## INTRODUCTION

Neuroblastoma (NB), the most common extracranial solid tumor derived from neural crest, accounts for approximately 15% of all pediatric cancer deaths [1]. NB is characterized by heterogeneous clinical behaviors, ranging from spontaneous regression to rapid progression or resistance to multimodal treatment, such as surgery, chemoradiotherapy, stem cell transplantation, and immunotherapy [1]. Better understanding the mechanisms underlying the progression and aggressiveness of NB is needed for improving the therapeutic efficiency. Matrix metalloproteinase 14 (MMP-14), also known as membrane type-1 matrix metalloproteinase, is able to degrade various extracellular matrix (ECM) components and facilitate the tumor cells to remodel and penetrate the ECM [2]. Previous studies have indicated that MMP-14 promotes the tumor invasion by functioning as a pericellular collagenase and an activator of proMMP-2, and is directly linked to tumorigenesis and aggressiveness [3]. In addition, MMP-14 promotes tumor angiogenesis through facilitating the transcription of vascular endothelial growth factor (*VEGF*) via activating the Src-tyrosine kinase pathway and increasing the cell surface localization of vascular endothelial growth factor receptor 2 [4–6]. Our previous studies have shown that MMP-14 is highly expressed in NB tissues and cell lines, and is correlated the aggressiveness and poor outcome of NB patients [7]. However, the regulatory mechanisms of MMP-14 expression in NB still remain largely unknown.

MicroRNAs (miRNAs), a class of small non-coding RNAs with 22 to 25 nucleotides in length, are able to inhibit gene expression at the post-transcriptional or translational levels through forming base pairs with their targets, usually in the 3′-untranslated region (3′-UTR) [8]. It has been established that miRNAs can participate in the tumorigenesis and aggressiveness [8]. For example, miR-203 inhibits the proliferation, migration, and invasion of glioma cells by disrupting the roundabout homolog 1/mitogen-activated protein kinase 1/matrix metalloproteinase 9 signaling axis [9]. miR-29b suppresses the growth, invasion, and metastasis of prostate cancer cells through repressing anti-apoptotic and pro-metastatic matrix molecules [10]. Previous studies have identified many aberrantly expressed miRNAs, which contribute to almost all aspects of tumor biology of NB, such as proliferation, apoptosis, differentiation, invasion, metastasis, and angiogenesis [11], suggesting the important roles of miRNAs in the progression and aggressiveness of NB.

In recent years, emerging studies show that endogenous miRNAs participate in the heterochromatin formation and regulation of gene transcription in human cells [12–15]. In the current study, through mining computational algorithm program and genome-wide Argonaute profiling dataset, we identified one binding site of miRNA-337-3p (miR-337-3p) within the *MMP-14* promoter, implicating its potential roles in the transcriptional control of *MMP-14* in NB. We demonstrate, for the first time, that miR-337-3p is under-expressed and anti-correlated with MMP-14 expression in clinical NB specimens, and directly binds the *MMP-14* promoter to suppress its transcription via inducing chromatin remodeling, thus inhibiting the growth, invasion, metastasis, and angiogenesis of NB cells *in vitro* and *in vivo*, suggesting the tumor suppressive roles of miR-337-3p in the progression of NB.

## RESULTS

### miR-337-3p is under-expressed and inversely correlated with MMP-14 levels in NB tissues and cell lines

**Figure 1:**
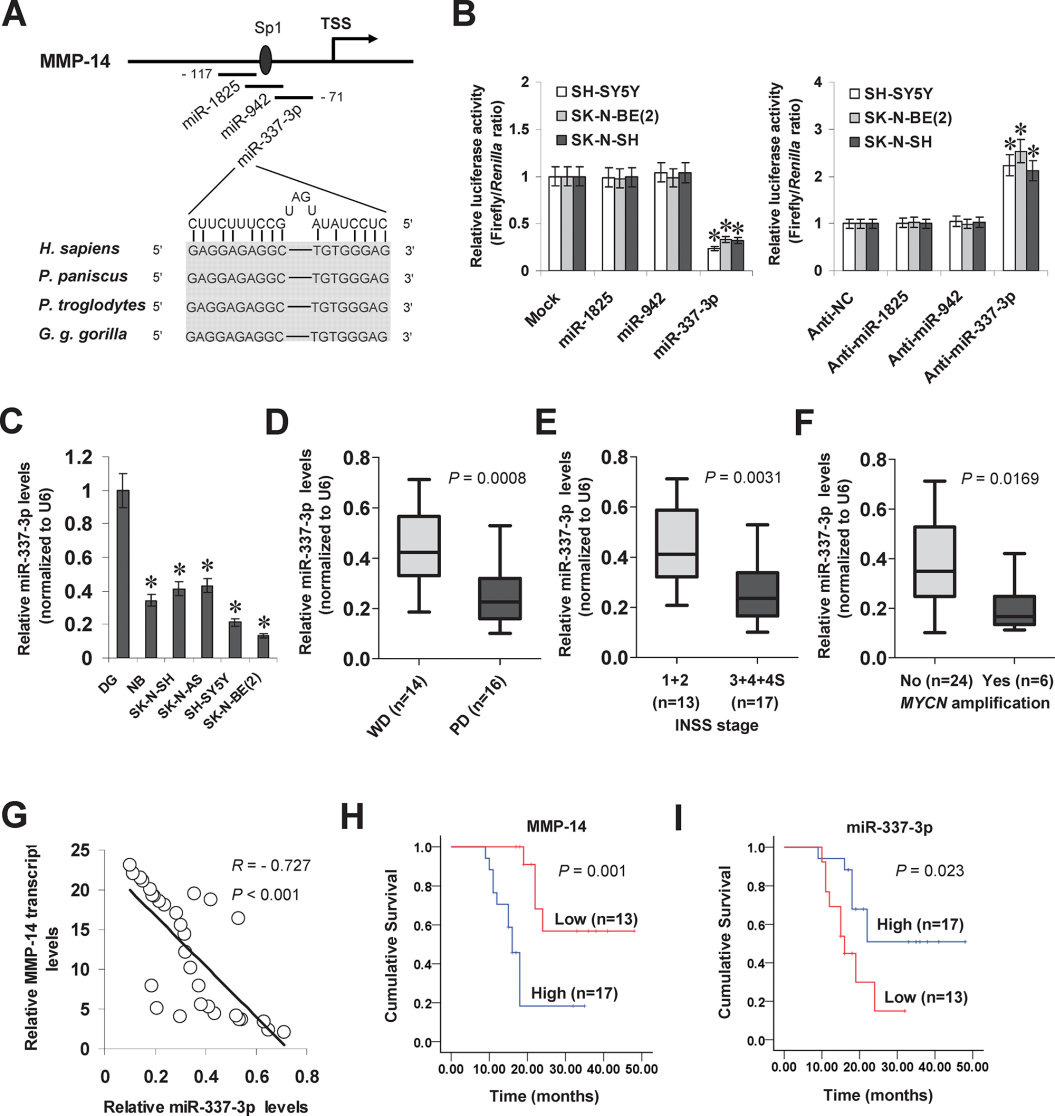
miR-337-3p is under-expressed and inversely correlated with MMP-14 levels in NB tissues and cell lines **A.** scheme of potential binding sites of miR-1825, miR-942, miR-337-3p, and Sp1 within the *MMP-14* promoter, locating at bases −117/–98, −106/–82, −90/–71, and −100/–95 relative to TSS. **B.** dual-luciferase assay showing the *MMP-14* promoter activity in SH-SY5Y, SK-N-BE(2), and SK-N-SH cells transfected with control mimic (mock), negative control inhibitor (anti-NC), and mimic or inhibitor (100 nmol/L) of miR-1825, miR-942, or miR-337-3p. **C.** real-time quantitative RT-PCR showing the miR-337-3p levels in normal dorsal ganglia (DG, *n* = 21), NB tissues (*n* = 30), and NB cell lines [SH-SY5Y, SK-N-SH, IMR-32, and SK-N-BE(2)]. **D.** real-time quantitative RT-PCR revealing the miR-337-3p levels in NB tissues with poor (PD) or well differentiation (WD). **E.** real-time quantitative RT-PCR showing the miR-337-3p levels in NB tissues with different INSS stages. **F.** real-time quantitative RT-PCR indicating the miR-337-3p levels in *MYCN*-amplified and *MYCN*-non-amplified NB tissues. **G.** the correlation between miR-337-3p expression and *MMP-14* transcript levels in NB tissues (*n* = 30). **H.** and **I.** Kaplan–Meier survival plots of 30 well-defined NB cases with high or low expression of MMP-14 or miR-337-3p. *P < 0.01 vs. mock, anti-NC, or DG.

To investigate the hypothesis that miRNA may participate in the transcription of *MMP-14* in NB, we searched the microPIR database [16] and genome-wide Argonaute profiling data (GSE40536). Enrichment of Argonaute 1 (AGO1) and Argonaute 2 (AGO2) was noted at bases −148/−68 relative to the transcription start site (TSS) of *MMP-14*. Within this region, there were potential binding sites of miR-1825, miR-942, and miR-337-3p with high complementarity, locating at bases −117/–98, −106/–82, and −90/–71 relative to the *MMP-14* TSS, respectively (Figure 1A). In addition, the binding site of specificity protein 1 (Sp1) was noted at −100/−95 bp region (Figure 1A). Dual-luciferase assay indicated that transfection of miRNA mimic and inhibitor of miR-337-3p, but not of miR-1825 or miR-942, resulted in altered promoter activity of *MMP-14* in cultured NB cell lines (Figure 1B). Mining the public Oncogenomics database (https://pob.abcc.ncifcrf.gov/cgi-bin/JK) revealed that the miR-337-3p host gene locus, locating within an imprinted region (chr14: 100397006–101488936) [17], was associated with copy number loss in NB tissues (Supplementary Figure S1A). RNA sequencing indicated that the miR-337-3p levels in NB tissues were inversely correlated with advanced international neuroblastoma staging system (INSS) stages (*P* = 0.0196), tumor progression (*P* = 0.0245), or *MYCN* amplification (*P* = 0.0365, Supplemenatry Figure S1B). In clinical tumor tissues derived from GEO datasets (http://www.ncbi.nlm.nih.gov/gds/), altered miR-337-3p levels were noted in some types of cancer, including up-regulation in breast cancer, glioblastoma, hepatocellular cancer, ovarian cancer, prostate cancer, and testicular tumor (Supplementary Figure S2A), and down-regulation in cervical cancer, colon cancer, pancreatic cancer, and renal cell carcinoma (Supplementary Figure S2B), suggesting the potential roles of miR-337-3p in tumorigenesis. In clinical NB and neuroblastic tumor specimens derived from the R2: microarray analysis and visualization platform (http://r2.amc.nl), the expression of MMP-14 was positively correlated with that of MYCN (correlation coefficient *R* = 0.261, *P* = 0.014; *R* = 0.317, *P* = 0.014), VEGF (*R* = 0.434, *P* < 0.0001; *R* = 0.485, *P* < 0.0001), or Sp1 (*R* = 0.405, *P* < 0.0001; *R* = 0.434, *P* = 0.0003, Supplemenatry Figure S1C). To further investigate the expression of miR-337-3p, real-time quantitative RT-PCR was applied to measure the mature miR-337-3p levels in normal dorsal ganglia, 30 NB specimens, and cultured SK-N-SH, SK-N-AS, SH-SY5Y, and SK-N-BE(2) cell lines. As shown in Figure 1C, miR-337-3p was under-expressed in the NB tissues and cell lines compared with normal dorsal ganglia. Lower miR-337-3p expression was observed in NB tissues with poor differentiation (*P* = 0.0008, Figure 1D), advanced INSS stage (*P* = 0.0031, Figure 1E), or *MYCN* amplification (*P* = 0.0169, Figure 1F). There was an inverse correlation between miR-337-3p expression and *MMP-14* transcript levels in NB tissues (*R* = – 0.727, *P* < 0.001, Figure 1G and Supplementary Table S1). Patients with high MMP-14 (*P* = 0.001) or low miR-337-3p (*P* = 0.023) expression had lower survival probability than those with low or high expression, respectively (Figure 1H and Figure 1I). These results indicated the under-expression of miR-337-3p in NB tissues and cell lines, which was inversely correlated with the *MMP-14* transcript levels.

### miR-337-3p inhibits the MMP-14 expression through transcriptional repression

**Figure 2:**
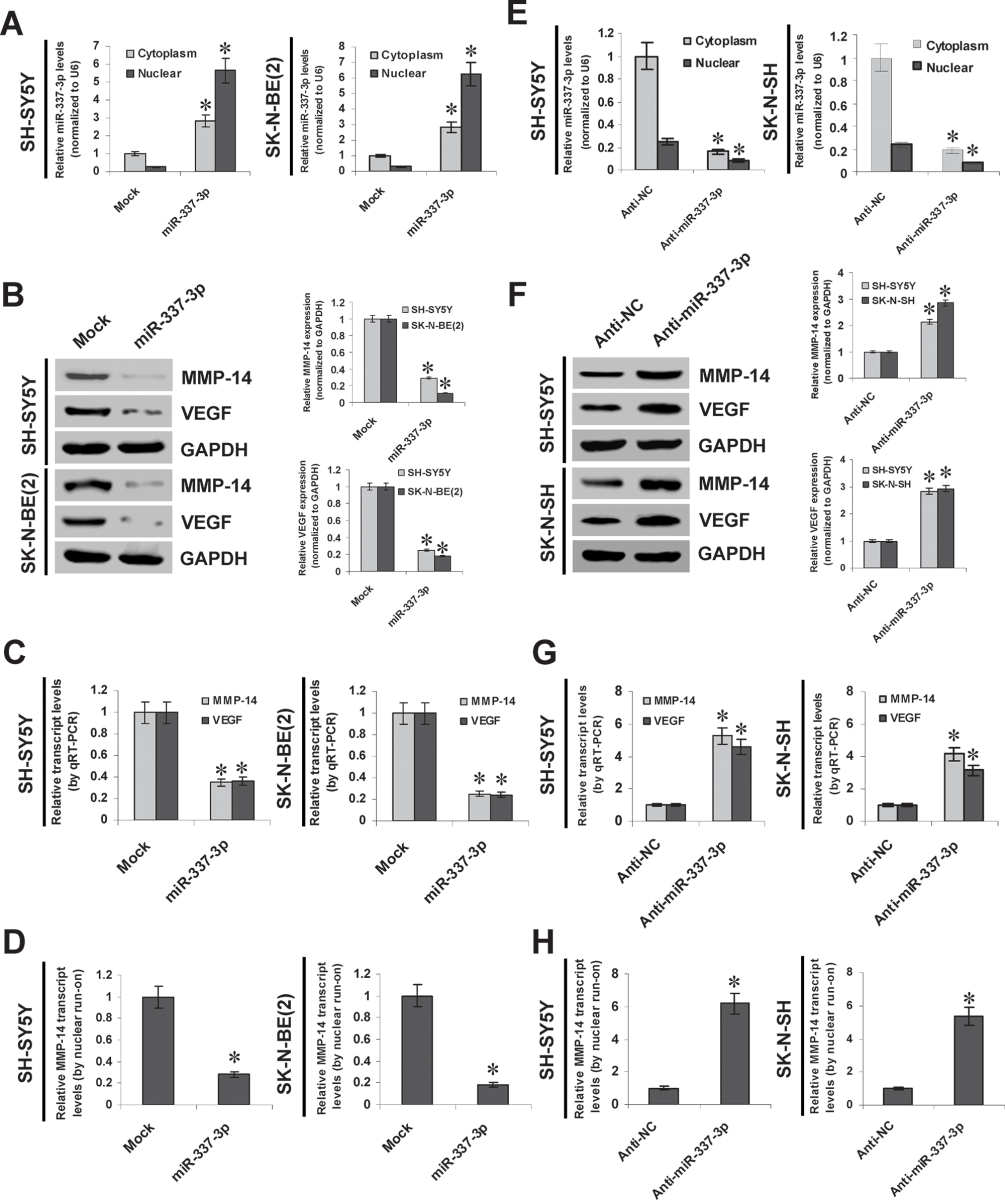
miR-337-3p inhibits the MMP-14 expression through transcriptional repression **A.** real-time quantitative RT-PCR showing the miR-337-3p levels in SH-SY5Y and SK-N-BE(2) cells stably transfected with empty vector (mock) or miR-337-3p precursor. **B.** and **C.** western blot and real-time quantitative RT-PCR showing the expression of MMP-14 and VEGF in SH-SY5Y and SK-N-BE(2) cells stably transfected with mock or miR-337-3p precursor. **D.** nuclear run-on assay indicating the nascent *MMP-14* transcript levels in NB cells stably transfected with mock or miR-337-3p precursor. **E.** real-time quantitative RT-PCR showing the miR-337-3p levels in SH-SY5Y and SK-N-SH cells transfected with negative control inhibitor (anti-N. 100 nmol/L) or anti-miR-337-3p inhibitor (100 nmol/L). **F.** and **G.** western blot and real-time quantitative RT-PCR showing the MMP-14 and VEGF levels in SH-SY5Y and SK-N-SH cells transfected with anti-NC (100 nmol/L) or anti-miR-337-3p inhibitor (100 nmol/L). **H.** nuclear run-on assay indicating the nascent *MMP-14* transcript levels in NB cells transfected with anti-NC (100 nmol/L) or anti-miR-337-3p inhibitor (100 nmol/L). *P < 0.01 vs. mock or anti-NC.

To explore the effects of miR-337-3p on MMP-14 expression in NB cell lines, we performed the miRNA over-expression experiments. Stable transfection of miR-337-3p precursor into SH-SY5Y and SK-N-BE(2) cells increased the cytoplasmic and nuclear miR-337-3p levels (Figure 2A). Western blot, real-time quantitative RT-PCR, and nuclear run-on assays demonstrated that stable over-expression of miR-337-3p decreased the protein and nascent transcript levels of MMP-14 in NB cells, than those stably transfected with empty vector (mock) (Figure 2B, Figure 2C, and Figure 2D). The expression levels of *VEGF*, the MMP-14 downstream target gene in NB [7], were significantly decreased in miR-337-3p over-expressing NB cells, consistent with the MMP-14 reduction (Figure 2B and Figure 2C). Since the analysis of microPIR database revealed no potential binding site of miR-337-3p within the *VEGF* promoter, we ruled out the possibility that miR-337-3p may directly suppress the transcription of *VEGF*. To further examine the suppressive roles of miR-337-3p in MMP-14 expression, we performed the miRNA knockdown experiments by transfection of anti-miR-337-3p or negative control (anti-NC) inhibitors into SH-SY5Y and SK-N-SH cells. Transfection of anti-miR-337-3p inhibitor obviously decreased the cytoplasmic and nuclear expression of miR-337-3p (Figure 2E), and increased the MMP-14 and VEGF protein levels than those of anti-NC-transfected cells (Figure 2F). Real-time quantitative RT-PCR and nuclear run-on assays indicated the enhanced transcriptional levels of *MMP-14* and *VEGF* in NB cells transfected with anti-miR-337-3p inhibitor, than those transfected with anti-NC (Figure 2G and Figure 2H). In addition, over-expression or knockdown of miR-337-3p in NB cells did not change the 3′-UTR luciferase activity of *MMP-14* and *VEGF* (Supplemenatry Figure S3A and Supplemenatry Figure S3B), indicating no involvement of post-transcription regulation by miR-337-3p. Moreover, knockdown of miR-337-3p resulted in no significant changes in the promoter activity and expression levels of MMP-14 in hepatocellular cancer HepG2 cells and prostate cancer PC-3 cells (Supplemenatry Figure S4A, Supplemenatry Figure S4B, Supplemenatry Figure S4C, and Supplemenatry Figure S4D). Meanwhile, ectopic expression of miR-337-3p repressed the promoter activity and transcription of *MMP-14* in cervical cancer HeLa cells, but not in renal cell carcinoma 786-O cells (Supplementary Figure S4E, Supplementary Figure S4F, Supplementary Figure S4G, and Supplementary Figure S4H). Overall, these results demonstrated that miR-337-3p considerably inhibited the MMP-14 expression through transcriptional repression.

### miR-337-3p recognizes the binding site and recruits AGO2 on *MMP-14* promoter

**Figure 3:**
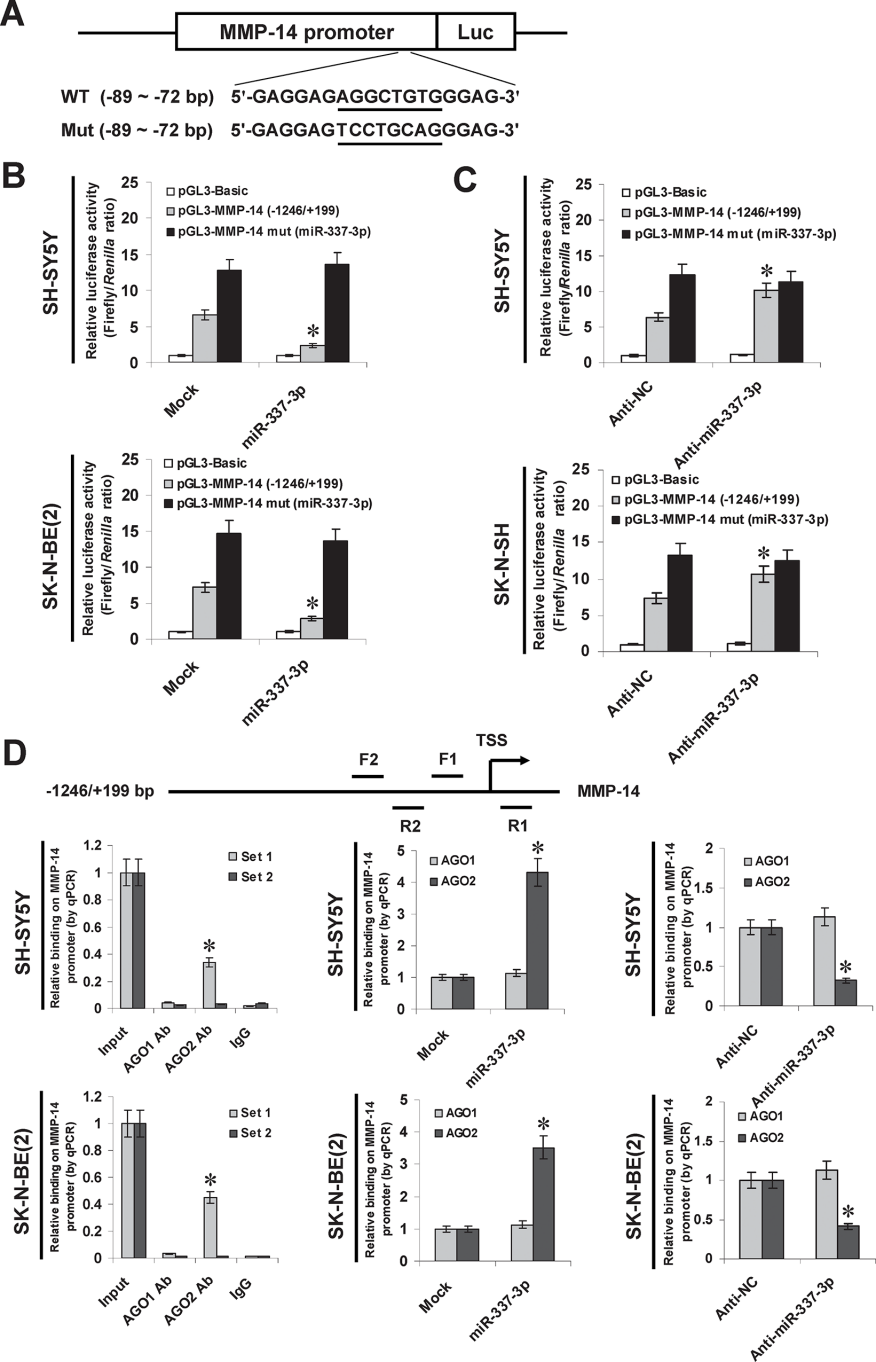
miR-337-3p recognizes the binding site and recruited AGO2 on MMP-14 promoter **A.** scheme and sequence of the intact miR-337-3p binding site (WT) and its mutation (Mut) within the *MMP-14* promoter-luciferase reporter vectors. **B.** dual-luciferase assay showing the activity of *MMP-14* promoter and its mutant in SH-SY5Y and SK-N-BE(2) cells stably transfected with empty vector (mock) or miR-337-3p precursor. **C.** dual-luciferase assay showing the activity of *MMP-14* promoter and its mutant in SH-SY5Y and SK-N-SH cells transfected with negative control inhibitor (anti-N. 100 nmol/L) or anti-miR-337-3p inhibitor (100 nmol/L). **D.** ChIP and qPCR assay indicating the enrichment of AGO1 and AGO2 on the *MMP-14* promoter in SH-SY5Y and SK-N-BE(2) cells transfected with mock, miR-337-3p precursor, anti-NC (100 nmol/L), or anti-miR-337-3p inhibitor (100 nmol/L). *P < 0.01 vs. mock or anti-NC.

To determine whether or not miR-337-3p could repress the MMP-14 expression by recognizing its binding site, the *MMP-14* promoter-luciferase reporter and a mutant of miRNA recognition site (Figure 3A) were transfected into NB cells stably transfected with empty vector (mock) or miR-337-3p precursor. The firefly luciferase activity normalized to that of *Renilla* was significantly reduced in the tumor cells stably transfected with miR-337-3p precursor (Figure 3B), and the effects were abolished by mutation of miR-337-3p binding site within the *MMP-14* promoter (Figure 3B). In addition, transfection of anti-miR-337-3p inhibitor increased the luciferase activity in SH-SY5Y and SK-N-SH cells (Figure 3C), while mutation of miR-337-3p recognition site abolished these effects (Figure 3C). Since previous studies have revealed the potential involvement of AGO1 and AGO2 in miRNA-induced transcriptional repression [12, 14, 18], the *MMP-14* promoter regions were assayed for enrichment of AGO1 and AGO2 by chromatin immunoprecipitation (ChIP) and real-time quantitative PCR (qPCR). In cultured SH-SY5Y and SK-N-BE(2) cells, enrichment of AGO2, but not of AGO1, was observed around the binding site (−122/+69) of miR-337-3p (Figure 3D). As controls, no *MMP-14* promoter regions were immunoprecipitated with unspecific antibody (isotype IgG) or detected by qPCR with primer set (−326/−157) distal to the binding site of miR-337-3p (Figure 3D). In addition, over-expression or knockdown of miR-337-3p increased and decreased the enrichment of AGO2 on *MMP-14* promoter, respectively (Figure 3D). These results indicated that miR-337-3p recognized the binding site and recruited AGO2 on the *MMP-14* promoter in NB cells.

### Involvement of AGO2 in miR-337-3p-induced epigenetic repression of *MMP-14* in NB cells

**Figure 4:**
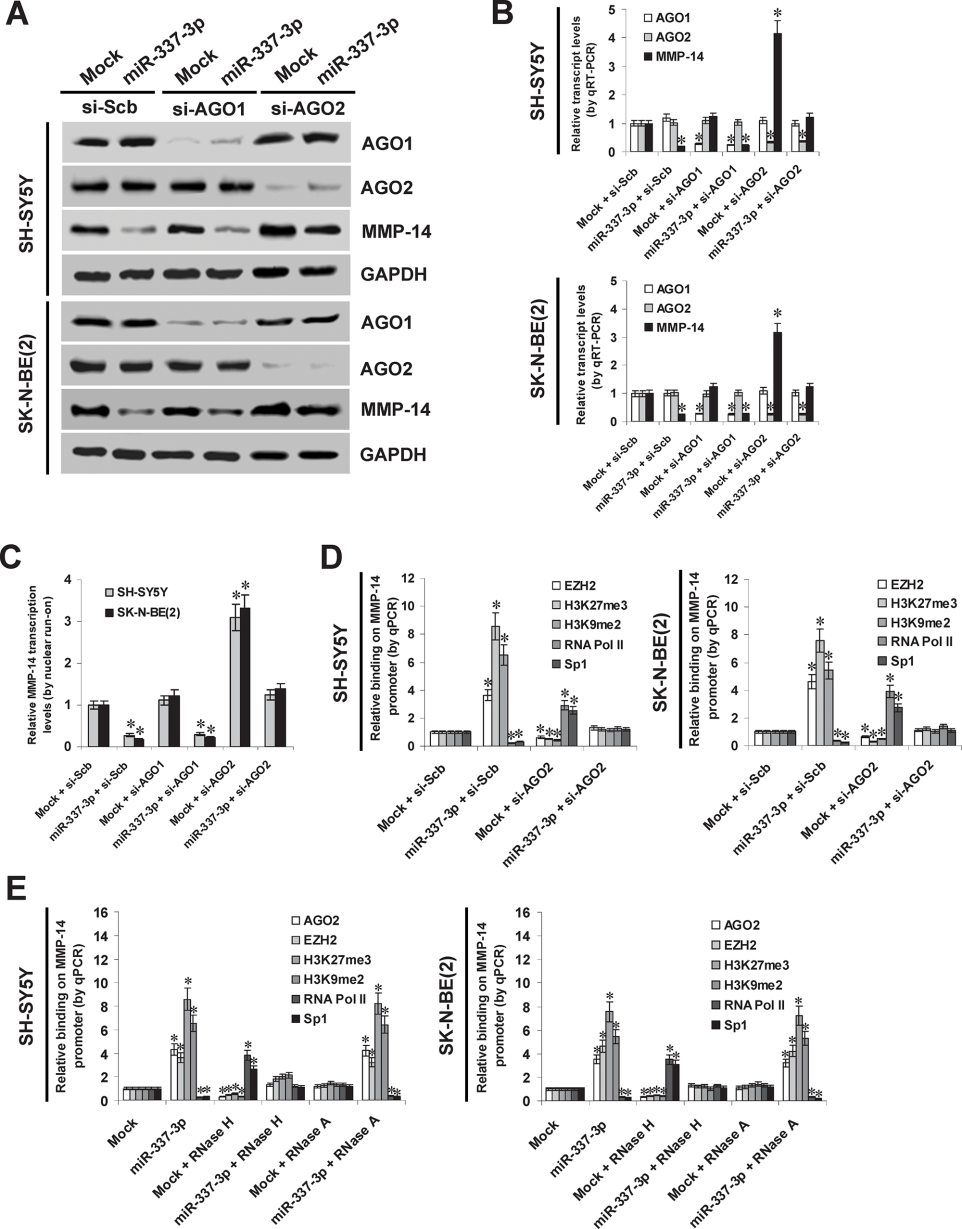
AGO2 is involved in miR-337-3p-induced epigenetic repression of MMP-14 in NB cells **A.** and **B.** western blot and real-time quantitative RT-PCR assays showing the expression of AGO1, AGO2, and MMP-14 in SH-SY5Y and SK-N-BE(2) cells transfected with empty vector (mock), miR-337-3p precursor, scramble siRNA (si-Scb), si-AGO1 (100 nmol/L), or si-AGO2 (100 nmol/L). **C.** nuclear run-on assay indicating the nascent *MMP-14* transcription in NB cells transfected with mock, miR-337-3p precursor, si-Scb, si-AGO1 (100 nmol/L), or si-AGO2 (100 nmol/L). **D.** ChIP and qPCR assay showing the binding of EZH2, H3K27me3, H3K9me2, RNA Pol II, and Sp1 on *MMP-14* promoter in NB cells stably transfected with mock or miR-337-3p precursor, and those co-transfected with si-Scb or si-AGO2 (100 nmol/L). **E.** ChIP and qPCR assay showing the enrichment of AGO2, EZH2, H3K27me3, H3K9me2, RNA Pol II, and Sp1 on *MMP-14* promoter in SH-SY5Y and SK-N-BE(2) stably transfected with mock or miR-337-3p, and those treated with RNase H or RNase A. *P < 0.01 vs. mock + si-Scb or mock.

To further investigate the potential roles of AGO1 and AGO2 in miR-337-3p-induced transcriptional repression of *MMP-14*, small interfering RNAs (siRNAs) against AGO1 and AGO2 were transfected into SH-SY5Y and SK-N-BE(2) cells stably transfected with empty vector (mock) or miR-337-3p precursor, respectively. Western blot, real-time quantitative RT-PCR, and nuclear run-on assays demonstrated that knockdown of AGO2, but not of AGO1, abolished the miR-337-3p-induced transcriptional repression of *MMP-14* in NB cells (Figure 4A, Figure 4B, and Figure 4C). In addition, administration of DNA methyltransferase inhibitor 5-aza-2′-deoxycytidine (5-Aza-CdR) resulted in no significant changes in miR-337-3p-induced repression of *MMP-14* in NB cells (Supplemenatry Figure S3C). Instead, stable transfection of miR-337-3p precursor into NB cells resulted in increased binding of epigenetic markers enhancer of zeste homolog 2 (EZH2), histone H3 lysine 27 trimethylation (H3K27me3), and histone H3 lysine 9 dimethylation (H3K9me2), and decreased binding of RNA polymerase II (RNA Pol II) on *MMP-14* promoter (Figure 4D), which was abolished by knockdown of AGO2 (Figure 4D). The binding of Sp1, a transcription factor essential for basal transcriptional activity of *MMP-14* promoter [19] (Supplemenatry Figure S5), was also abolished by ectopic expression of miR-337-3p into NB cells (Figure 4D). To explore whether miR-337-3p directly binds the *MMP-14* promoter, lysates from miR-337-3p over-expressing NB cells were pretreated with RNase H or RNase A. As shown in Figure 4E, RNase H treatment, but not RNase A treatment, prevented the NB cells from increased enrichment of AGO2, EZH2, H3K27me3, and H3K9me2, and decreased binding of RNA Pol II and Sp1 on the *MMP-14* promoter induced by miR-337-3p, indicating the direct binding of miR-337-3p on *MMP-14* promoter. Collectively, these results indicated the involvement of AGO2 in miR-337-3p-induced epigenetic repression of *MMP-14* in NB cells.

### miR-337-3p suppresses the growth, invasion, metastasis, and angiogenesis of NB cells through repressing MMP-14 *in vitro*

**Figure 5:**
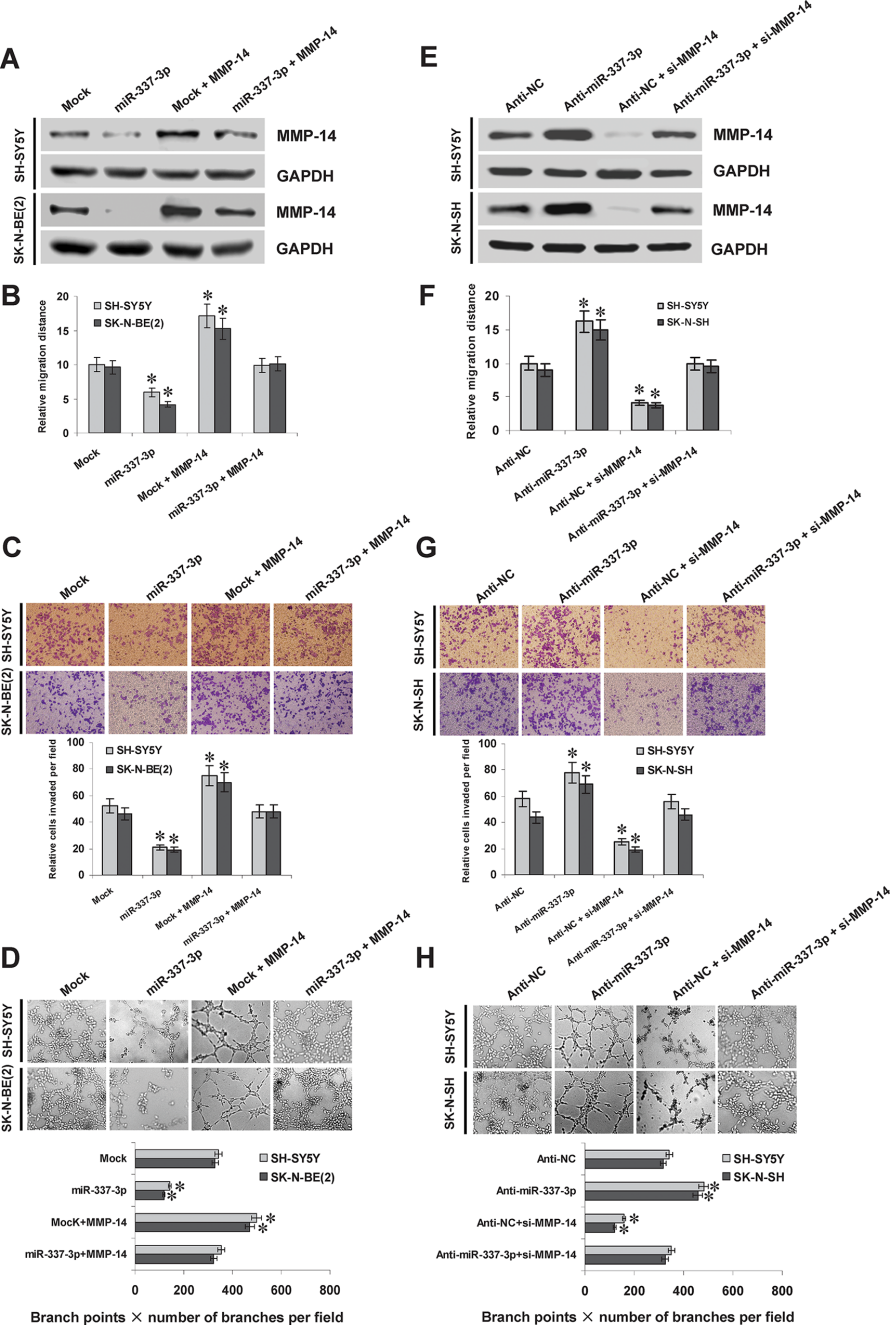
miR-337-3p suppresses the migration, invasion, and angiogenesis of NB cells *in vitro* **A.** and **E.** western blot showing the MMP-14 expression in NB cells transfected with empty vector (mock), miR-337-3p precursor, negative control inhibitor (anti-NC, 100 nmol/L), or anti-miR-337-3p inhibitor (100 nmol/L), and those co-transfected with *MMP-14* or si-MMP-14 (100 nmol/L). **B.** and **F.** quantification of scratch migration assay showing the migration of NB cells transfected with mock, miR-337-3p precursor, anti-NC (100 nmol/L), or anti-miR-337-3p inhibitor (100 nmol/L), and those co-transfected with *MMP-14* or si-MMP-14 (100 nmol/L). **C.** and **G.** representation (top) and quantification (bottom) of matrigel invasion assay showing the invasion capability of NB cells transfected with mock, miR-337-3p precursor, anti-NC (100 nmol/L), or anti-miR-337-3p inhibitor (100 nmol/L), and those co-transfected with *MMP-14* or si-MMP-14 (100 nmol/L). **D.** and **H.** representation (top) and quantification (bottom) of tube formation assay showing the angiogenic capability of NB cells transfected with mock, miR-337-3p precursor, anti-NC (100 nmol/L), or anti-miR-337-3p inhibitor (100 nmol/L), and those co-transfected with *MMP-14* or si-MMP-14 (100 nmol/L). *P < 0.01 vs. mock or anti-NC.

Since previous studies indicate that MMP-14 promotes the growth, migration, invasion, and angiogenesis of tumor cells [7], we further investigated the effects of miR-337-3p over-expression and MMP-14 restoration on cultured NB cells. Western blot indicated that transfection of *MMP-14* rescued the miR-337-3p-repressed MMP-14 expression (Figure 5A). In MTT colorimetric assay, tumor cells stably transfected with miR-337-3p precursor possessed the decreased cell viability capability, when compared with those stably transfected with empty vector (mock; Supplemenatry Figure S6A). In scratch migration assay, miR-337-3p over-expression attenuated the migration capability of SH-SY5Y and SK-N-BE(2) cells (Figure 5B and Supplemenatry Figure S6B). Matrigel invasion assay revealed that NB cells stably transfected with miR-337-3p precursor presented an impaired invasion capacity than mock cells (Figure 5C). The tube formation of endothelial cells was suppressed by treatment with the medium preconditioned by stable transfection of NB cells with miR-337-3p precursor (Figure 5D). In addition, transfection of *MMP-14* into SH-SY5Y and SK-N-BE(2) cells restored the decrease in growth, migration, invasion, and angiogenesis induced by stable over-expression of miR-337-3p (Figure 5B, Figure 5C, Figure 5D, Supplementary Figure S6A, and Supplementary Figure S6B). Interestingly, ectopic expression of miR-337-3p also attenuated the growth, invasion, and angiogenesis of cervical cancer HeLa cells, which was rescued by restoration of MMP-14 expression (Supplementary Figure S6C, Supplementary Figure S6D, Supplemenatry Figure S6E, and Supplementary Figure S6F). On the other hand, we examined the effects of miR-337-3p knockdown on NB cells. Introduction of anti-miR-337-3p inhibitor into SH-SY5Y and SK-N-SH cells resulted in enhanced MMP-14 expression (Figure 5E), and increased abilities in cell viability (Supplementary Figure S6G), migration (Figure 5F and Supplementary Figure S6H), invasion (Figure 5G), and angiogenesis (Figure 5H). In addition, restoration of MMP-14 expression via transfection of si-MMP-14 rescued the SH-SY5Y and SK-N-SH cells from their changes in these biological features induced by knockdown of miR-337-3p (Figure 5F, Figure 5G, Figure 5H, Supplementary Figure S6G, and Supplementary Figure S6H). These results indicated that miR-337-3p remarkably decreased the growth, migration, invasion, and angiogenesis of NB cells through repressing MMP-14 *in vitro*.

### Over-expression of miR-337-3p attenuates the growth, metastasis, and angiogenesis of NB cells *in vivo*

**Figure 6:**
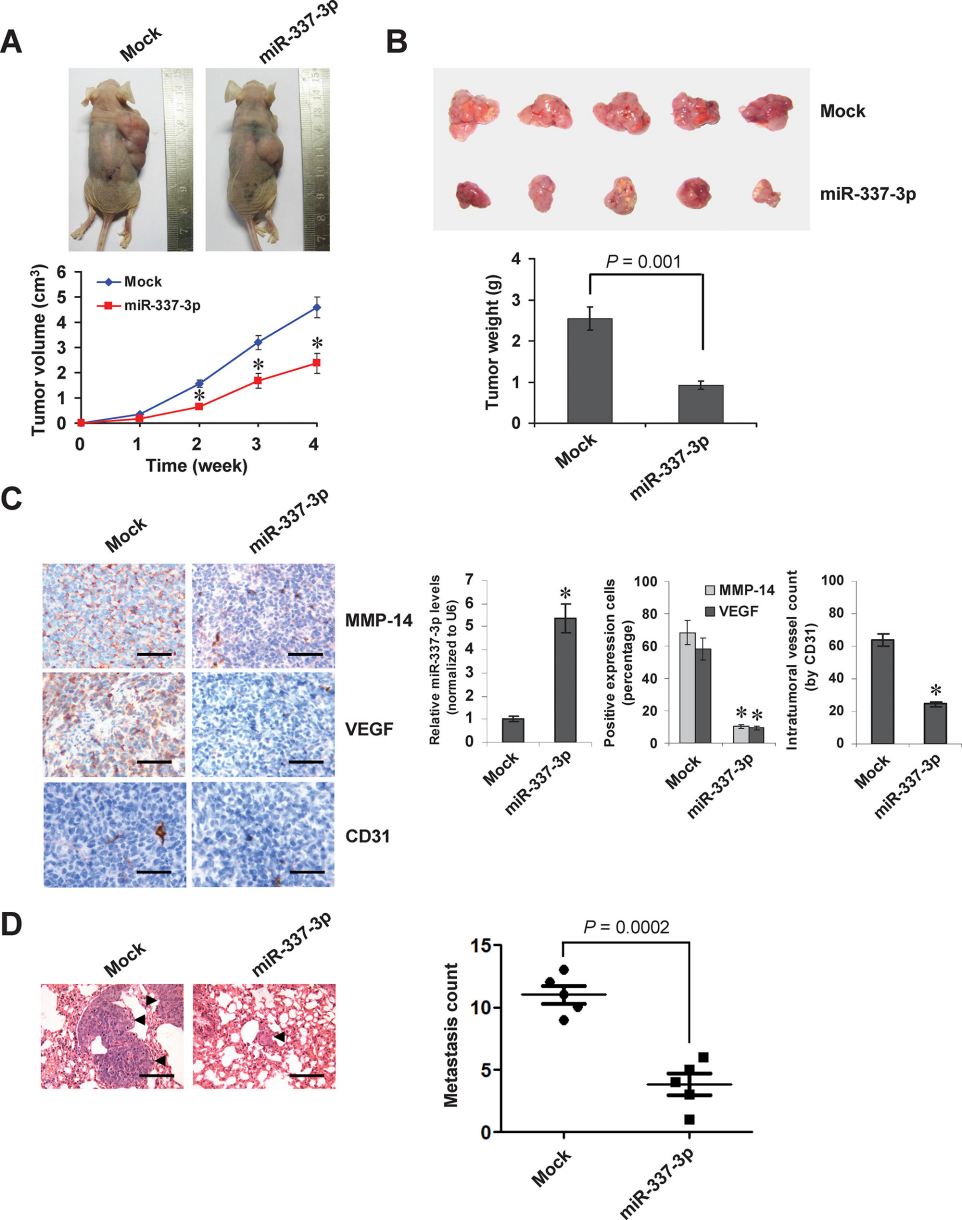
Over-expression of miR-337-3p attenuates the growth and metastasis of NB cells *in vivo* **A.** tumor growth curve of SH-SY5Y cells (1 × 10^6^) stably transfected with empty vector (mock) or miR-337-3p precursor in athymic nude mice (*n* = 5 for each group), after hypodermic injection for 4 weeks. **B.** representation (top) and quantification (bottom) of xenograft tumors formed by hypodermic injection of SH-SY5Y cells stably transfected with mock or miR-337-3p precursor. **C.** immunohistochemical staining and real-time quantitative RT-PCR showing the expression of MMP-14, VEGF, CD31, and miR-337-3p within tumors formed by hypodermic injection of SH-SY5Y cells stably transfected with mock or miR-337-3p precursor. Scale bars: 100 μm. **D**. representation (left, arrowhead) and quantification (right) of lung metastasis after injection of SH-SY5Y cells (0.4 × 10^6^) stably transfected with mock or miR-337-3p precursor into the tail vein of athymic nude mice (*n* = 5 for each group). Scale bars: 100 μm. *P < 0.001 vs. mock.

We next investigated the efficacy of miR-337-3p against tumor growth and metastasis *in vivo*. Stable transfection of miR-337-3p precursor into SH-SY5Y cells resulted in decreased growth and tumor weight of subcutaneous xenograft tumors in athymic nude mice, when compared to those stably transfected with empty vector (mock) (Figure 6A and Figure 6B). In addition, the miR-337-3p levels within tumors were increased, and the intratumoral expression of MMP-14 and VEGF was also reduced by stable transfection of miR-337-3p precursor (Figure 6C). Moreover, stable transfection of miR-337-3p precursor resulted in decrease in CD31-positive microvessels and mean vessel density within tumors (Figure 6C). In the experimental metastasis studies, SH-SY5Y cells stably transfected with miR-337-3p precursor established statistically fewer lung metastatic colonies than mock group (Figure 6D). These results suggested that miR-337-3p could inhibit the growth, metastasis, and angiogenesis of NB cells *in vivo*.

## DISCUSSION

Canonically, miRNAs inhibit gene expression at the post-transcriptional levels, and this process is executed through recognition of miRNA–mRNA pairing by RNA-induced silencing complex machinery, with AGO proteins as the main effectors [11]. Recent evidence shows that a subset of miRNAs predominantly localizing in the nucleus of human cells can recognize complementary genomic sites within gene promoters, and participate in the regulation of gene expression [12, 14]. At promoter complementary sites, miRNAs can function as an interface between chromatin remodeling complexes and genome [20, 21]. For example, endogenous miR-709 is able to repress the transcription of early growth response 2 through forming epigenetic silencing complexes with H3K27me3 and AGO1 on its promoter [18]. miR-320 induces the transcriptional repression of RNA polymerase III polypeptide D (*POLR3D*) through directing the association of AGO1, EZH2, and H3K27me3 with *POLR3D* promoter [14]. Meanwhile, miR-423-5p-induced transcriptional repression of progesterone receptor (*PR*) is associated with the recruitment of AGO2 to an antisense non-coding RNA transcribed from *PR* promoter, and accompanied by an increase in H3K9me2 enrichment at *PR* promoter [12]. However, studies on the roles of miRNAs in regulating gene transcription are still in its infancy, and it is currently urgent to investigate the expression and functions of these miRNAs in the tumor biology of NB.

miR-337-3p is a novel identified miRNA involved in the tumorigenesis. Previous studies show that miR-337-3p is under-expressed in endometrial cancer [22], gastric cancer [23], colon cancer [24], and medulloblastomas [25]. Loss of miR-337-3p expression is associated with the lymph node metastasis of gastric cancer [23]. miR-337-3p promotes the senescence of colorectal cancer cells by repressing the expression of casein kinase II subunit alpha [26], and sensitizes the lung cancer cell lines to paclitaxel by directly repressing the expression of signal transducer and activator of transcription 3 and Ras-related Protein 1A [27]. Since miR-337-3p host gene resides within the chromosome 14q32.2 [28], an imprinted region essential for development [17], and aberrant regulation of miRNAs located on chromosome 14q32 is involved in tumorigenesis [24, 28–30], we suspect that miR-337-3p may also participate in the progression of NB. In this study, we found the under-expression of miR-337-3p in NB tissues, which was associated with unfavorable outcome of patients. Meanwhile, in line with previous studies [31], we noticed the copy number loss of chromosome 14q32.2 in NB tissues, implying that under-expression of miR-337-3p may be due to allelic loss, which warrants our further investigation. Through an integrative approach to analyze the public datasets, we identified miR-337-3p as a novel repressor of *MMP-14* transcription. Our evidence showed that miR-337-3p suppressed the expression of MMP-14 through binding its promoter and inducing transcriptional repression in NB cells. The fact that restoration of MMP-14 was sufficient to prevent the NB cells from miR-337-3p-inhibited biological behaviors indicates that miR-337-3p exerts its tumor suppressive functions, at least in part, through repressing the MMP-14 expression in NB. In addition, we noted the different expression profiles of miR-337-3p in publicly available clinical tumor datasets, and found that miR-337-3p attenuated the MMP-14 expression in cervical cancer cells, but not in hepatocellular cancer, prostate cancer, or renal cell carcinoma cells. Our evidence shows that miR-337-3p suppresses the aggressiveness of cervical cancer cells, suggesting the tissue-specific expression patterns and functions of miR-337-3p in human tumors.

Human *MMP-14* gene, consisting of 10 exons, is localized at chromosome 14q11 [19]. Previous studies show that Sp1 is essential for maintaining the *MMP-14* transcription [19]. Transcriptionally inactive *MMP-14* gene promoter is characterized by deposition of H3K27me3 and hypermethylation of CpG islands [32], while low levels of H3K27me3 and under-methylation are observed at the *MMP-14* promoter region in invasive and migratory cancer cells [32], suggesting that histone modification and DNA methylation are crucial factors impacting the transcriptional efficiency of *MMP-14* promoter. Since our preliminary data revealed that administration of DNA methyltransferase inhibitor resulted in no significant changes in miR-337-3p-induced transcriptional repression of *MMP-14* in NB cells, we ruled out the possible involvement of aberrant promoter hypermethylation in this process. In this study, we found that in miR-337-3p over-expressing NB cells, the recruitment of repressive epigenetic markers EZH2, H3K27me3, and H3K9me2 was increased, accompanied by decreased enrichment of RNA Pol II and Sp1 on *MMP-14* promoters. These findings indicate that miR-337-3p may induce epigenetic inactivation by deposition of repressive chromatin marks, or through a process that prevents RNA Pol II stalling at gene promoter.

The mechanisms underlying the recruitment of miRNAs to accessible promoter sequences include direct binding to promoter [18] and indirect association with promoter-derived non-coding transcripts [12, 13]. Since RNase H treatment (specifically degrades the RNA present in RNA-DNA hybrid) [12] attenuated the miR-337-3p-induced enrichment of AGO2, EZH2, H3K27me3, and H3K9me2, these findings indicated the direct binding of miR-337-3p on *MMP-14* promoter. Previous studies have implicated the participation of AGO1 and AGO2 in small RNA-inhibited gene expression [33]. AGO1, but not AGO2, was selectively enriched in vicinity to the miR-744 complementary site on *cyclin B1* promoter [34]. In this study, our evidence indicated that AGO2, but not AGO1, was enriched at the *MMP-14* promoter in miR-337-3p over-expressing NB cells. In addition, knockdown of *AGO2* abolished the miR-337-3p-induced binding of repressive epigenetic markers on *MMP-14* promoter. We believe that miR-337-3p/AGO2 complexes may bring in co-repressors such as histone methyltransferases to repress gene expression, which warrants our further investigation.

In summary, we have shown that miR-337-3p is under-expressed in human NB, and over-expression of miR-337-3p inhibits the growth, invasion, metastasis, and angiogenesis of NB cells *in vitro* and *in vivo*. Furthermore, miR-337-3p suppresses the transcription of *MMP-14* via epigenetic repression of its promoter activity in NB cell lines. This study extends our knowledge about the regulation of *MMP-14* at transcriptional level by miRNAs, and suggests that miR-337-3p may be of potential values as a novel therapeutic target for human NB.

## MATERIALS AND METHODS

### Patient tissue samples

Approval to conduct this study was obtained from the Institutional Review Board of Tongji Medical College (approval number: 2011-S085). Fresh tumor specimens from 30 well-established primary NB cases were collected, and stored at −80°C until use. Based on the Shimada classification system, including the mitosis karyorrhexis index, degree of neuroblastic differentiation and stromal maturation, and patient's age, 14 patients were classified as having favorable histology and 16 as having unfavorable histology. According to the INSS, 4 patients were classified as stage 1, 9 as stage 2, 9 as stage 3, 4 as stage 4, and 4 as stage 4S. Total RNA of normal human dorsal ganglia, pooling from 21 male/female Caucasians, was obtained from Clontech (Mountain View, CA).

### miRNA prediction and expression detection

miRNA binding sites within the *MMP-14* promoter were analyzed using the algorithm microPIR [16]. Cytoplasmic and nuclear fractions were prepared by using the NE-PER Nuclear and Cytoplasmic Extraction Reagents (Thermo Fisher Scientific, Inc., Waltham, MA). The mature miR-337-3p levels were determined using Bulge-Loop^TM^ miRNAs qPCR Primer Set (RiboBio Co. Ltd, Guangzhou, China). After cDNA was synthesized with a miRNA-specific stem-loop primer, the quantitative PCR was performed with the specific primers (Supplementary Table S2). The miRNA levels were normalized as to those of U6 snRNA.

### Western blot

Cellular protein was extracted with 1 × cell lysis buffer (Promega, Madison, WI). Western blot was performed as previously described [7, 21, 35–40], with antibodies specific for MMP-14 (Abcam Inc, Cambridge, MA), VEGF (Santa Cruz Biotechnology, Santa Cruz, CA), AGO1 (Cell Signaling Technology, Inc., Danvers, MA), AGO2 (Cell Signaling Technology, Inc.), Sp1 (Santa Cruz Biotechnology), and glyceraldehyde-3-phosphate dehydrogenase (GAPDH, Santa Cruz Biotechnology).

### Real-time quantitative RT-PCR

Total RNA was isolated with RNeasy Mini Kit (Qiagen Inc., Valencia, CA). The reverse transcription reactions were conducted with Transcriptor First Strand cDNA Synthesis Kit (Roche, Indianapolis, IN). Real-time PCR was performed with SYBR Green PCR Master Mix (Applied Biosystems, Foster City, CA) and primers indicated in Supplemenatry Table S2. The transcript levels were analyzed by 2^−△△Ct^ method.

### Cell culture and transfection

Human NB cell lines SK-N-SH (HTB-11), SK-N-AS (CRL-2137), SH-SY5Y (CRL-2266), and SK-N-BE(2) (CRL-2271), hepatocellular cancer cell line HepG2 (HB-8065), prostate cancer cell line PC-3 (CRL-1435), cervix cancer cell line HeLa (CCL-2), renal cell carcinoma cell line 786-O (CRL-1932), and umbilical vein endothelial cells (HUVEC, CRL-1730) were purchased from American Type Culture Collection (Rockville, MD). Cell lines were authenticated by the provider, used within 6 months after resuscitation of frozen aliquots, and grown in RPMI1640 medium (Life Technologies, Inc., Gaithersburg, MD) supplemented with 10% fetal bovine serum (Life Technologies, Inc.), penicillin (100 U/ml), and streptomycin (100 μg/ml). Cells were maintained at 37°C in a humidified atmosphere of 5% CO_2_ and applied for transfection or treatment with 5-Aza-CdR (Sigma, St. Louis, MO) as indicated. Anti-miR-337-3p or negative control inhibitors (RiboBio Co. Ltd) were transfected into confluent cells with Lipofectamine 2000 (Life Technologies, Inc.).

### pre-miRNA construct and stable transfection

According to the pre-miR-337-3p (5′-GAACGGCTT CATACAGGAGTT-3′) sequence documented in the miRNA Registry database [41], oligonucleotides encoding the precursor of miR-337-3p (Table S3) were subcloned into pcDNA3.1(−) (Genechem Co., Ltd, Shanghai, China). The plasmids pcDNA3.1 and pcDNA3.1-miR-337-3p were transfected into tumor cells, and stable cell lines were screened by administration of neomycin (Invitrogen, Carlsbad, CA).

### Luciferase reporter assay

Human *MMP-14* promoter luciferase reporter constructs, and *MMP-14* and *VEGF* 3′-UTR luciferase reporter vectors were previously described [7, 39, 42]. Mutation of miR-337-3p binding site was established with GeneTailor^TM^ Site-Directed Mutagenesis System (Invitrogen) and PCR primers (Supplemenatry Table S3). Dual-luciferase assay was performed as previously described [7, 33, 35, 36]. For *MMP-14* promoter and 3′-UTR activity, the luciferase signal was normalized by firefly/*Renilla* and *Renilla*/firefly ratio, respectively.

### Nuclear run-on assay

Nuclear run-on assay was performed based on the incorporation of biotin-16-uridine-5′-triphosphate (biotin-16-UTP) into nascent transcripts as previously described [33]. Briefly, nuclei of 5 × 10^6^ tumor cells were isolated and consequently incubated in reaction buffer containing rNTPs and biotin-16-UTP (Roche, Indianapolis, IN) at 30°C for 45 min. The reaction was stopped by adding RNase-free DNase I, and nuclei were lysed and treated with proteinase K. Total RNA was extracted using Trizol (Invitrogen), and biotinylated nascent RNA was purified using agarose-conjugated streptavidin beads (Invitrogen) for real-time quantitative RT-PCR assay.

### Gene over-expression and knockdown

Human *MMP-14* expression vector was previously described [7]. To restore the miRNA-repressed MMP-14 expression, stable cell lines were transfected with the recombinant vector pcDNA3.1-MMP14. Human *Sp1* cDNA (2358 bp) was amplified from NB tissues (Supplemenatry Table S3) and subcloned into pcDNA3.1 (Invitrogen). The 21-nucleotide siRNAs against the encoding region of *MMP-14* [7], *AGO1* [33], and *AGO2* [33] were chemically synthesized (RiboBio Co. Ltd) and transfected with Genesilencer Transfection Reagent (Genlantis, San Diego, CA). The scramble siRNA (si-Scb) was applied as a control (Supplemenatry Table S3).

### Chromatin immunoprecipitation

ChIP assay was performed according to the instructions of EZ-ChIP kit (Upstate Biotechnology, Temacula, CA) [33, 36, 40, 43], with antibodies for AGO1, AGO2, EZH2, H3K27me3, H3K9me2, RNA Pol II, and Sp1 (Upstate Biotechnology, Temacula, CA). Lysates were treated with either RNase H (10 U) or RNase A (20 μg) prior to immunoprecipitation. DNA was sonicated into fragments of an average size of 200 bp. Real-time qPCR was performed with SYBR Green PCR Master Mix and primer sets indicated in Supplemenatry Table S2. The amount of immunoprecipitated DNA was calculated in reference to a standard curve and normalized to input DNA.

### Cell viability assay

Tumor cells were cultured in 96-well plates at 5 × 10^3^ cells per well. Cell viability was monitored by the 2- (4, 5-dimethyltriazol-2-yl)-2, 5-diphenyl tetrazolium bromide (MTT, Sigma) colorimetric assay [33]. All experiments were done with 6–8 wells per experiment and repeated at least three times.

### Scratch migration assay

Tumor cells were cultured in 24-well plates and scraped with the fine end of 1-ml pipette tips (time 0). Plates were washed twice with phosphate buffered saline to remove detached cells, and incubated with the complete growth medium. Cell migration was photographed using 10 high-power fields, at 0, 24 hr post-induction of injury. Remodeling was measured as diminishing distance across the induced injury, normalized to the 0 hr control, and expressed as outgrowth (μm) [7, 35, 36, 43].

### Cell invasion assay

Matrigel invasion assay was performed using membranes coated with Matrigel matrix (BD Science, Sparks, MD). Homogeneous single cell suspensions (1 × 10^5^ cells/well) were added to the upper chambers and allowed to invade for 24 hrs at 37°C in a CO_2_ incubator. Invaded cells were stained with 0.1% crystal violet for 10 min at room temperature. Quantification of invaded cells was performed according to published criteria [7, 35, 37, 38, 43, 44].

### Tube formation assay

Fifty microliters of growth factor-reduced matrigel were polymerized on 96-well plates. HUVECs were serum starved in RPMI1640 medium for 24 hrs, suspended in RPMI1640 medium preconditioned with tumor cells, added to the matrigel-coated wells at the density of 5 × 10^4^ cells/well, and incubated at 37°C for 18 hrs. Quantification of anti-angiogenic activity was calculated as previously described [35–37].

### *In vivo* growth and metastasis assay

All animal experiments were approved by the Animal Care Committee of Tongji Medical College (approval number: Y20080290). For the *in vivo* tumor growth studies, 2-month-old male nude mice (*n* = 5 per group) were injected subcutaneously in the upper back with 1 × 10^6^ tumor cells stably transfected with empty or miR-337-3p precursor vectors. One month later, mice were sacrificed and examined for tumor weight and gene expression. The experimental metastasis (0.4 × 10^6^ tumor cells per mouse, *n* = 5 per group) studies were performed with 2-month-old male nude mice as previously described [7, 35, 36].

### Immunohistochemistry

Immunohistochemical staining was performed as previously described [7, 35, 36, 45], with antibodies specific for MMP-14 (Abcam Inc; Santa Cruz Biotechnology; 1:200 dilution), VEGF, and CD31 (Santa Cruz Biotechnology; 1:200 dilutions). The negative controls included parallel sections treated without primary antibody or with rabbit polyclonal IgG (Abcam Inc.). The immunoreactivity in each tissue section was assessed by at least two pathologists. The degree of positivity was determined according to the percentage of positive tumor cells.

### Statistical analysis

Unless otherwise stated, all data were shown as mean ± standard error of the mean (SEM). The χ^2^ analysis and Fisher exact probability analysis were applied to compare the gene expression in tumor tissues with different clinicopathological features. Pearson's coefficient correlation was applied for analyzing the relationship among gene expression. The Kaplan-Meier method was used to estimate survival rates, and the log-rank test was used to assess survival difference. Difference of tumor cells was determined by *t* test or analysis of variance (ANOVA).

## SUPPLEMENTARY FIGURES AND TABLES


