# Supplementary material for: miRNA-337-3p suppresses neuroblastoma progression by repressing the transcription of matrix metalloproteinase 14
Source: Oncotarget. 2015 Jun 15;6(26):22452–66. doi: 10.18632/oncotarget.4311 (PMC4673175; doi:10.18632/oncotarget.4311)
Supplement: Supplementary file 1 [file oncotarget-06-22452-s001.pdf]

SUPPLEMENTARY FIGURES AND TABLES

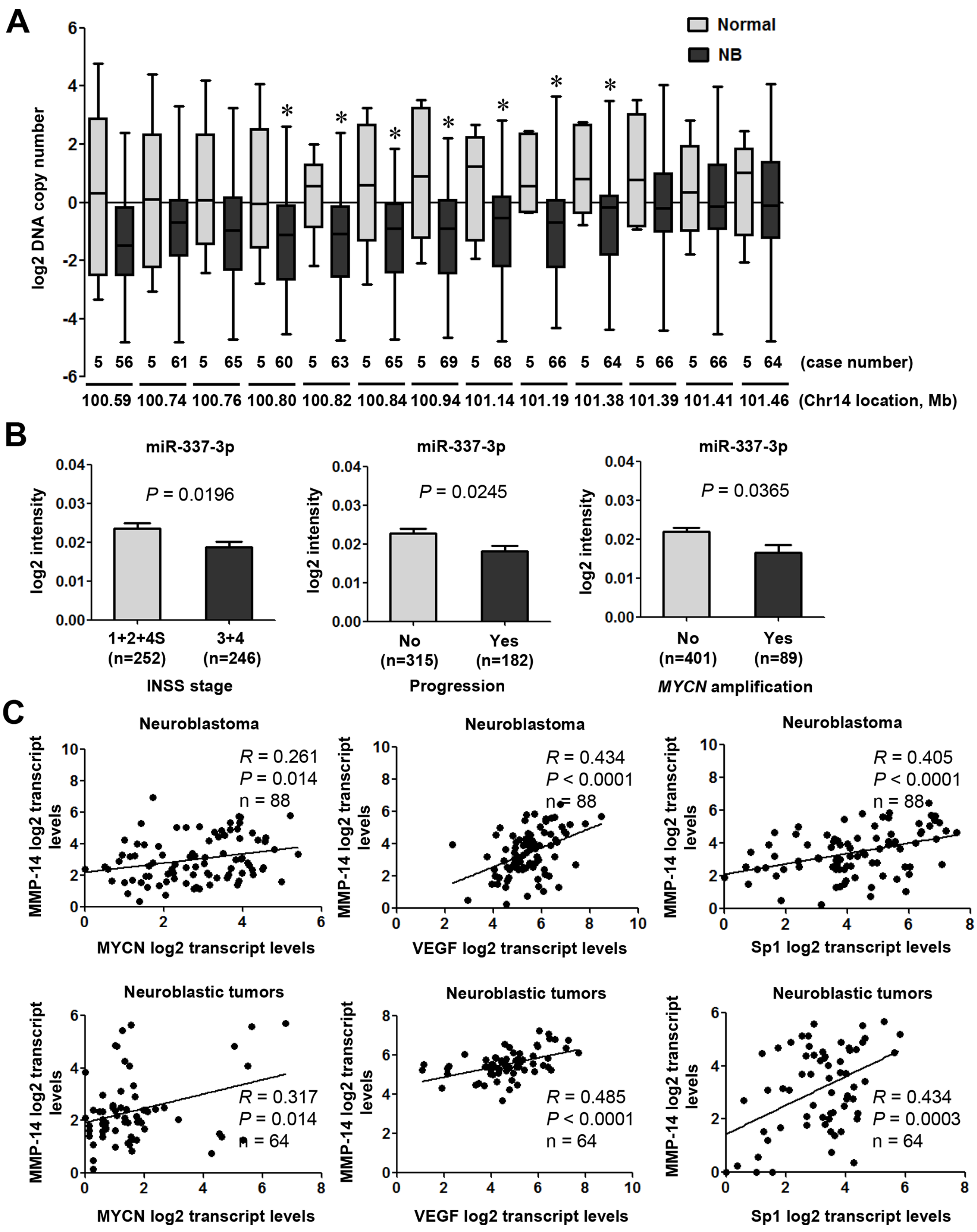

Supplementary Figure S1: Data mining of miR-337-3p and MMP-14 expression in public databases. A. the copy number loss of miR-337-3p host gene locus, locating at chr14: 101340830-101340922, in NB tissues derived from Oncogenomics database (https://pob.abcc.ncifcrf.gov/cgi-bin/JK). **B.** RNA-Seq data (GSE49710) revealing the miR-337-3p levels in NB tissues with different INSS stages, tumor progression, or *MYCN* amplification status. **C.** the positive correlation between MMP-14 expression and MYCN, VEGF, or Sp1 levels in clinical NB and neuroblastic tumor specimens derived from the R2: microarray analysis and visualization platform (http://r2.amc.nl). *$P < 0.01$ vs. normal genomic DNA.

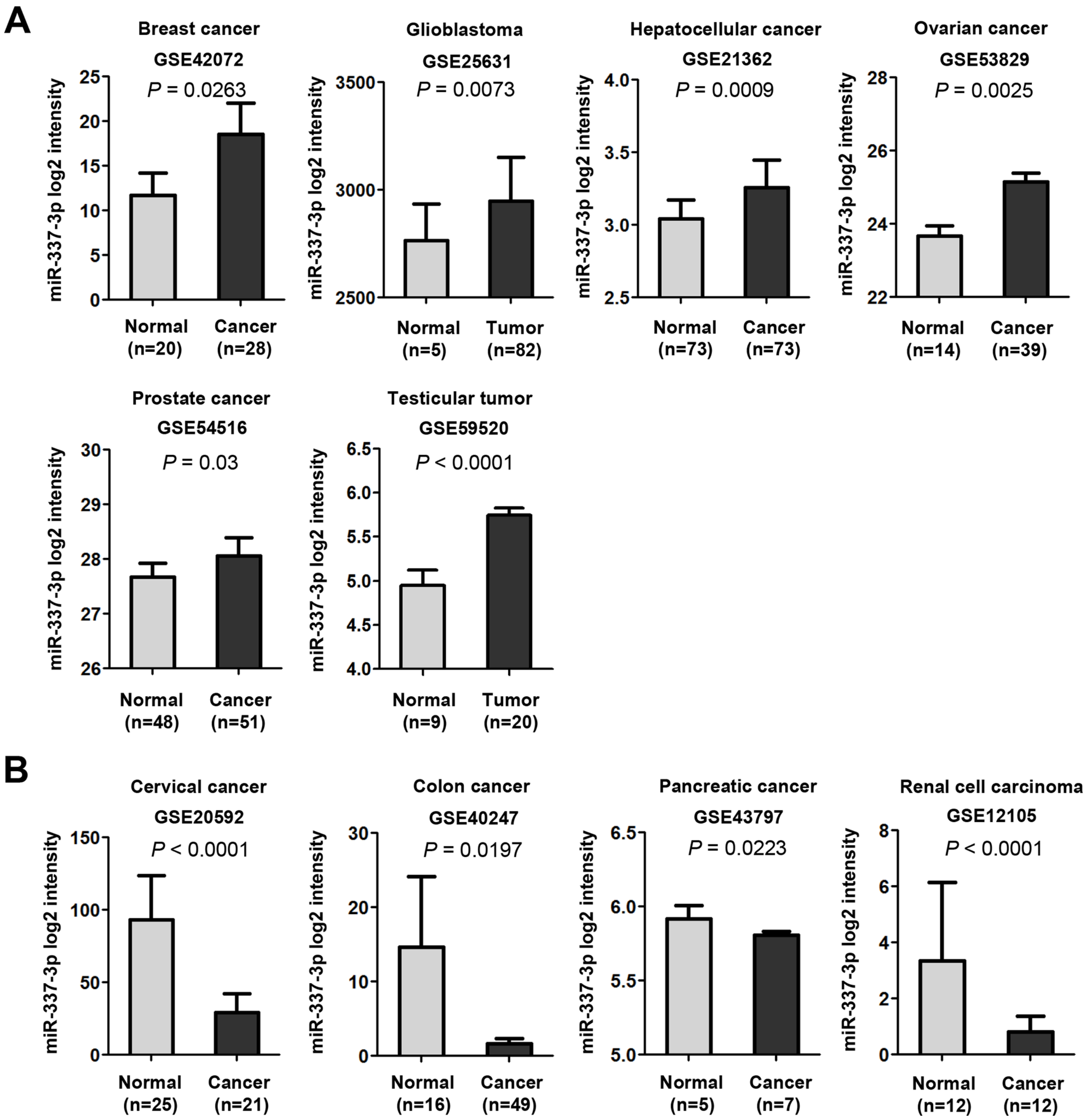

Supplementary Figure S2: Expression profiles of miR-337-3p in human cancer tissues. A. the analysis of GEO datasets (http://www.ncbi.nlm.nih.gov/gds/) showing the high miR-337-3p levels in breast cancer, glioblastoma, hepatocellular cancer, ovarian cancer, prostate cancer, and testicular tumor tissues, than their corresponding normal tissues. **B.** the analysis of GEO datasets revealing the under-expression of miR-337-3p in cervical cancer, colon cancer, pancreatic cancer, and renal cell carcinoma tissues, than their corresponding normal tissues.

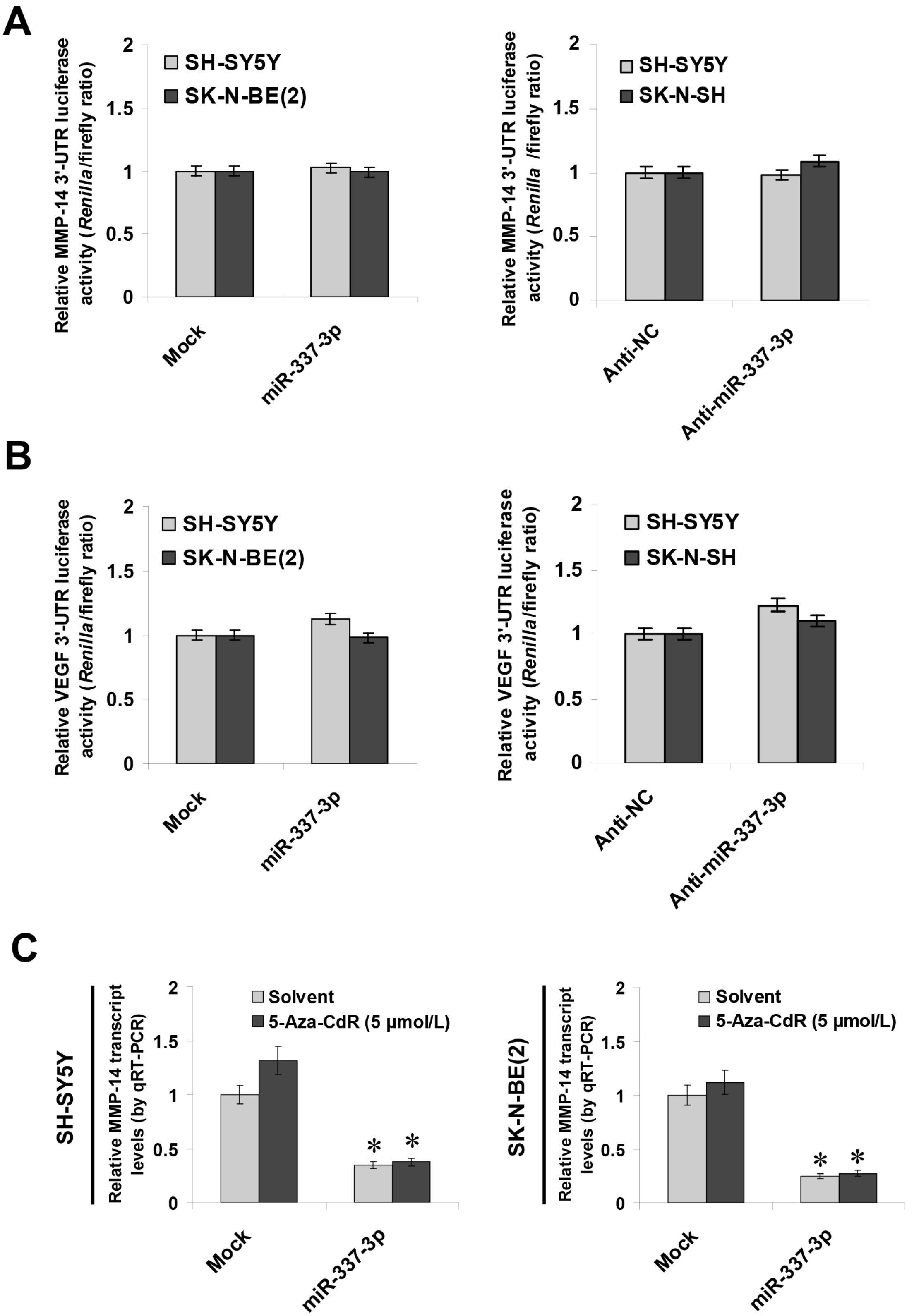

Supplementary Figure S3: Effects of miR-337-3p on the 3′-UTR activity of *MMP-14* and *VEGF*. A. and **B.** Dual-luciferase reporter assay showing the 3′-UTR activity of *MMP-14* and *VEGF* in SH-SY5Y, SK-N-BE(2), and SK-N-SH cells transfected with empty vector (mock), miR-337-3p precursor, negative control inhibitor (anti-NC, 100 nmol/L), or anti-miR-337-3p inhibitor (100 nmol/L). **C.** Real-time quantitative RT-PCR showing the MMP-14 levels in SH-SY5Y and SK-N-BE(2) cells stably transfected with mock or miR-337-3p precursor, and those treated with 5-Aza-CdR (5 μmol/L) for 24 hrs. * $P < 0.01$ vs. mock.

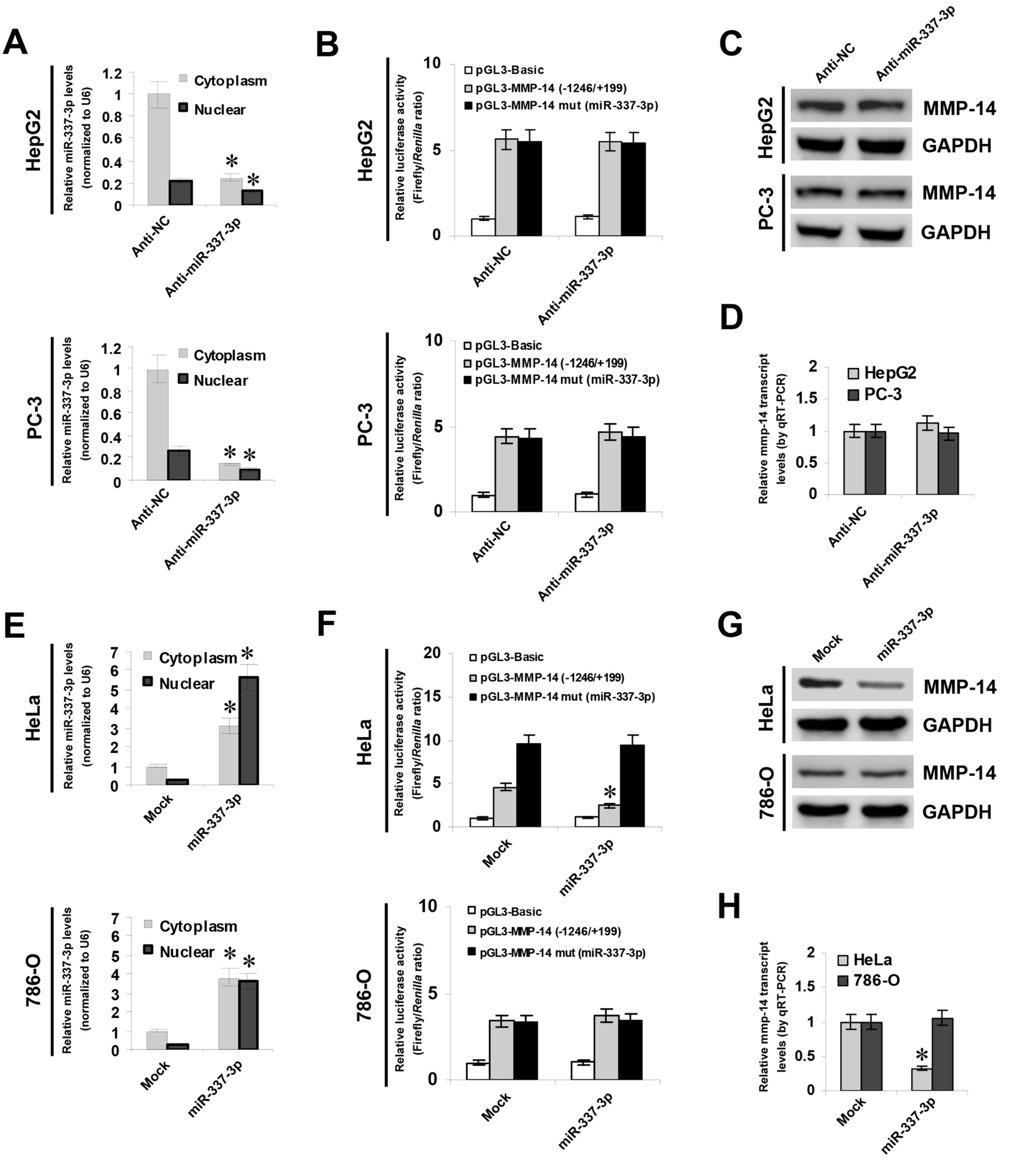

Supplementary Figure S4: Effects of miR-337-3p on the promoter activity and expression of MMP-14 in human cancer cells. Cancer cell lines were transfected with negative control inhibitor (anti-NC, 100 nmol/L), anti-miR-337-3p inhibitor (100 nmol/L), empty vector (mock), or miR-337-3p precursor. Real-time quantitative RT-PCR **A.** and **E.** dual-luciferase assay **B.** and **F.** western blot **C.** and **G.** and real-time quantitative RT-PCR **D.** and **H.** showing the miR-337-3p levels, *MMP-14* promoter activity, MMP-14 protein and transcript levels in cancer cells, respectively. * $P < 0.01$ vs. anti-NC or mock.

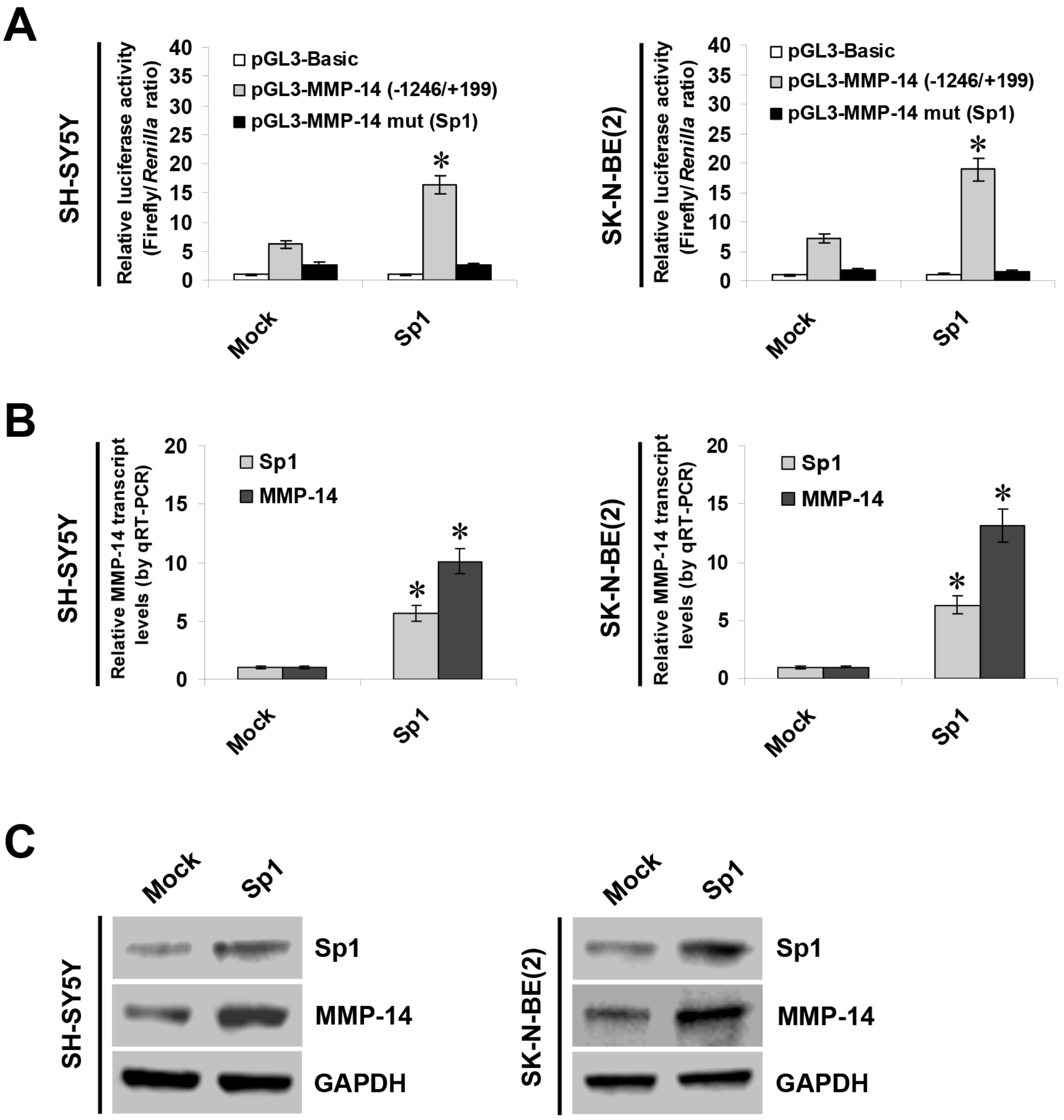

Supplementary Figure S5: Sp1 is essential for MMP-14 expression in NB cells. A. dual-luciferase assay showing the activity of *MMP-14* promoter and its mutant in SH-SY5Y and SK-N-BE(2) cells transfected with empty vector (mock) or *Sp1*. **B.** and **C.** real-time quantitative RT-PCR and western blot assays showing the transcript and protein levels of Sp1 and MMP-14 in NB cells transfected with mock or *Sp1*. * $P < 0.01$ vs. mock.

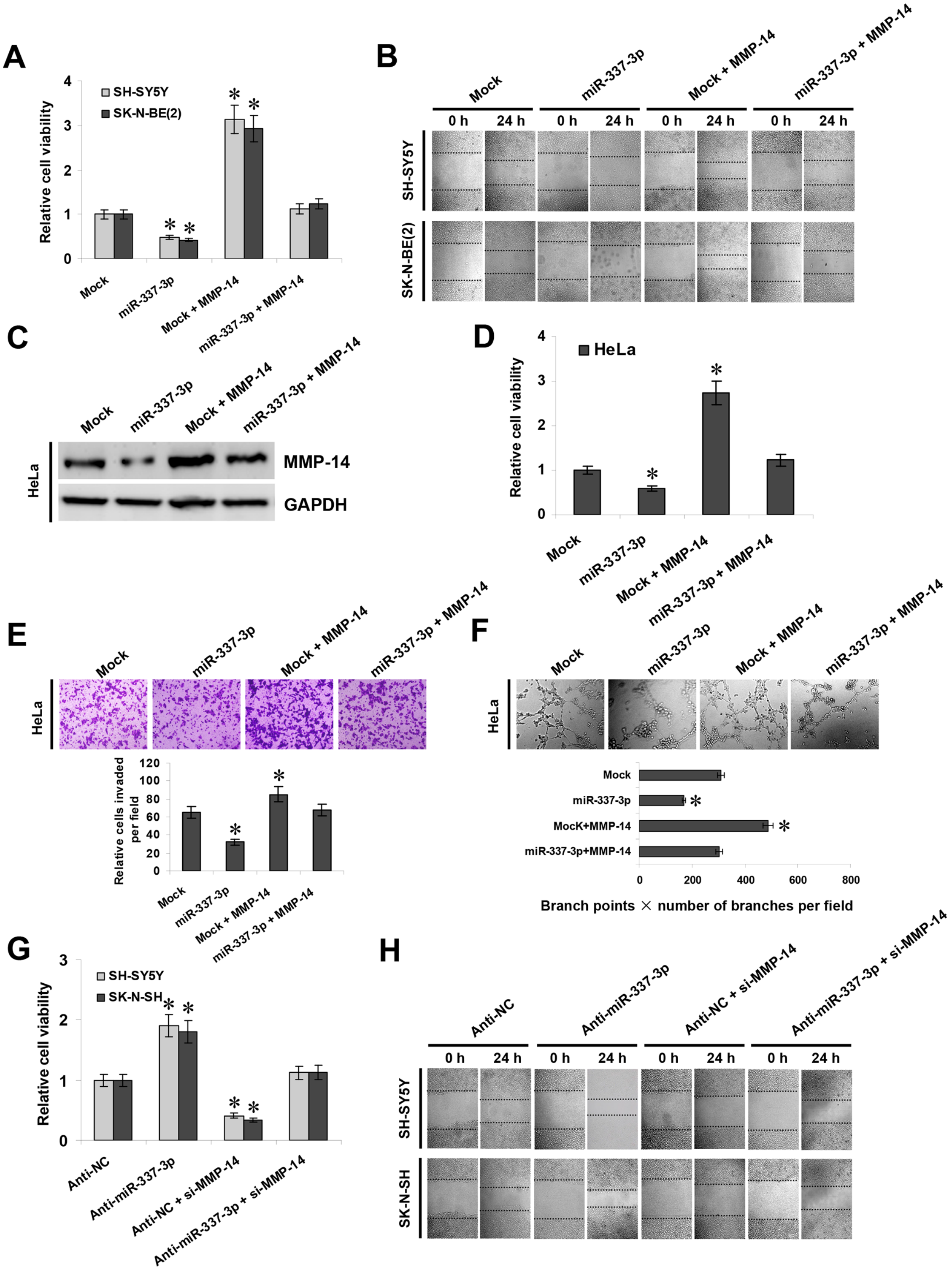

Supplementary Figure S6: Restoration of MMP-14 expression rescues the effects of miR-337-3p on tumor cells. The *MMP-14* or si-MMP-14 (100 nmol/L) was transfected into NB or HeLa cells transfected with empty vector (mock), miR-337-3p precursor, negative control inhibitor (anti-NC, 100 nmol/L), or anti-miR-337-3p inhibitor (100 nmol/L). The MTT colorimetric assay **A.** and **G.** and representation of scratch assay **B.** and **H.** showing the viability and migration capability of NB cells, respectively. Western blot **C.** indicating the MMP-14 expression levels in HeLa cells. The MTT colorimetric assay **D.** representation and quantification of matrigel invasion assay **E.** and tube formation assay **F.** showing the viability, invasion, and angiogenesis capability of HeLa cells, respectively. * $P < 0.01$ vs. mock or anti-NC.

Supplementary Table S1: Expression profiles of miR-337-3p and MMP-14 in NB specimens

Patient No.	Age (months)	Gender	*MYCN* amplification	INSS stage	Histology	miR-337-3p expression*	MMP-14 transcript levels*
1	2	F	No	4	PD	0.321	12.154
2	1	F	No	3	PD	0.318	14.431
3	10	M	No	2	PD	0.302	15.561
4	5	M	No	3	PD	0.529	16.411
5	12	F	No	3	PD	0.285	17.121
6	3	F	No	3	PD	0.236	18.112
7	4	M	No	2	PD	0.216	18.562
8	18	F	Yes	3	PD	0.421	18.782
9	16	M	No	4	PD	0.195	19.231
10	24	M	Yes	3	PD	0.191	19.312
11	3	M	No	4S	PD	0.356	23.121
12	4	M	No	2	WD	0.712	2.122
13	8	M	No	2	WD	0.648	2.321
14	18	F	No	1	WD	0.632	3.421
15	16	M	No	2	WD	0.543	3.656
16	14	F	No	1	WD	0.534	3.721
17	5	M	No	4S	WD	0.298	4.012
18	12	M	No	4S	WD	0.522	4.123
19	3	M	No	2	WD	0.435	4.432
20	2	M	No	2	WD	0.208	5.121
21	7	F	Yes	3	PD	0.181	19.542
22	2	M	Yes	3	PD	0.152	20.121
23	1	M	Yes	4	PD	0.142	21.123
24	1	M	Yes	4	PD	0.113	21.542
25	4	M	No	4S	PD	0.101	22.112
26	3	F	No	2	WD	0.412	5.243
27	2	M	No	1	WD	0.381	5.531
28	2	M	No	1	WD	0.372	7.892
29	4	M	No	3	WD	0.186	7.921
30	1	M	No	2	WD	0.342	10.231

M, male; F, female; PD, poorly differentiated; WD; well differentiated; *, normalized to normal dorsal ganglia

Supplementary Table S2: Primer sets used for qRT-PCR and ChIP

Primer set	Primers	Sequence	Product size (bp)	Application
MMP14	Forward	5′-GCCTTCTGTTCCTGATAA-3′	225	qRT-PCR
	Reverse	5′-CCATCCTTCCTCTCGTAG-3′		
VEGF	Forward	5′-GGCAGAATCATCACGAAG-3′	276	qRT-PCR
	Reverse	5′-TGTGCTGTAGGAAGCTCA-3′		
AGO1	Forward	5′-CAGCAGGGGATGGGAAAAAA-3′	178	qRT-PCR
	Reverse	5′-GGCTTGAAACGGGTGGACTT-3′		
AGO2	Forward	5′-TTTCAAGGACAGGCACAA-3′	102	qRT-PCR
	Reverse	5′-TTACAGACCTCCAGGGGAT-3′		
Sp1	Forward	5′-CTGCCGCTCCCAACTTAC-3′	220	qRT-PCR
	Reverse	5′-TTGCCTCCACTTCCTCGA-3′		
GAPDH	Forward	5′-AGAAGGCTGGGGCTCATTTG-3′	258	qRT-PCR
	Reverse	5′-AGGGGCCATCCACAGTCTTC-3′		
miR-337-3p	Forward	RiboBio		qRT-PCR
	Reverse	RiboBio		
U6	Forward	RiboBio		qRT-PCR
	Reverse	RiboBio		
MMP-14 set 1	Forward	5′-CAACCAGGAAAGGAGGGC-3′	191	ChIP
(–122/+69)	Reverse	5′-TCGGCTTGGAGTTAAAGG-3′		
MMP-14 set 2	Forward	5′-TCAAGCCACTCAGAATATGC-3′	170	ChIP
(–326/–157)	Reverse	5′-ACCAAGAACTGGAAGGAAAA-3′		

MMP-14, matrix metalloproteinases 14; VEGF, vascular endothelial growth factor; AGO1, argonaute 1; AGO2, argonaute 2; Sp1, specificity protein 1; GAPDH, glyceraldehyde 3-phosphate dehydrogenase

Supplementary Table S3: Oligonucleotide sets used for constructs and small interfering RNAs

RNAs	Sequences
Pre-miR-337-3p	5′-TGCTGCTCCTATATGATGCCTTTCTTCGTTTTGGCCACTGACTGACGAAGA AAGGCATCATATAGGAG-3′ (sense);
	5′-CCTGCTCCTATATGATGCCTTTCTTCGTCAGTCAGTGGCCAAAACGAAGAA AGGCATCATATAGGAGC-3′ (antisense)
Pre-miR-NC	5′-TGCTGAAATGTACTGCGCGTGGAGACGTTTTGGCCACTGACTGACGTCTC CACGCAGTACATTT-3′ (sense);
	5′-CCTGAAATGTACTGCGTGGAGACGTCAGTCAGTGGCCAAAACGTCTCCAC GCGCAGTACATTTC-3′ (antisense)
pGL3-MMP14 mut	5′-CTGGGGCGGGGACGGAGGAGCAATTGTGTTCAAAGGGAGGGA-3′ (sense);
(miR-337-3p)	5′-CTCCTCCGTCCCCGCCCCAGTGCCCTCCTT-3′ (antisense)
pcDNA3.1-MMP14	5′-CGCCCAAGCTTATGTCTCCCGCCCCAAG-3′ (sense);
	5′-CTAGTCTAGATCAGACCTTGTCCAGCA-3′ (antisense)
pcDNA3.1-Sp1	5′-ACGGGCCCTCTAGACTCGAGATGAGCGACCAAGATCACTCC-3′ (sense);
	5′-TTAAACTTAAGCTTGGTACCTCAGAAGCCATTGCCACTGATATTAATG-3′ (antisense)
Anti-NC	RiboBio
Anti-miR-337-3p	RiboBio
si-Scb	5′-GAACGAUCGAGUAAACGGAtt-3′ (sense);
	5′-UCCGUUUACUCGAUCGUUCtt-3′ (antisense)
si-AGO1	5′-GAGAAGAGGUGCUCAAGAAtt-3′ (sense);
	5′-UUCUUGAGCACCUCUUCUCtt-3′ (antisense)
si-AGO2	5′-GCACGGAAGUCCAUCUGAAtt-3′ (sense);
	5′-UUCAGAUGGACUUCCGUGCtt-3′ (antisense)
si-MMP14	5′-CCAGAAGCUGAAGGUAGAAtt-3′ (sense);
	5′-UUCUACCUUCAGCUUCUGGtt-3′ (antisense)

MMP-14, matrix metalloproteinases 14; Pre-miR-NC, negative control pre-miRNA; Sp1, specificity protein 1; Anti-NC, negative control inhibitor; si-Scb, scramble siRNA; AGO1, argonaute 1; AGO2, argonaute 2
